# 

**Published:** 1887-04

**Authors:** 


### CONTENTS.

COMMUNICATIONS.

Irregularities........................................................................ 161
A Ten Minutes Sermon on Nutrition.............................................. 169
The Relation of Dentistry and Medicine......................................... 175
New Remedies........................................................................ 179
Mississippi Valley Association of Dental Surgeons........................ 181
Minutes of Mississippi Valley Association of Dental Surgeons........ 192

LEGISLATION.

An Act to Regulate the Practice of Dentistry in Alabama............. 199
The Dental Law of Indiana as Amended in 1887.......................... 201

SELECTIONS.

Surgical Aphorisms.................................................................. 204
A New Method for the Restoration of Respiration Lost under Chloroform.................................................................. 207
Lister's Latest Antiseptic.......................................................... 208
SALMAGUNDI........................................................................... 210

EDITORIAL.

A Well Deserved Honor.............................................................. 212

